# Antiglutamate Receptor Antibodies and Cognitive Impairment in Primary Antiphospholipid Syndrome and Systemic Lupus Erythematosus

**DOI:** 10.3389/fimmu.2016.00005

**Published:** 2016-02-01

**Authors:** Maria Gerosa, Barbara Poletti, Francesca Pregnolato, Gabriella Castellino, Annalisa Lafronza, Vincenzo Silani, Piersandro Riboldi, Pier Luigi Meroni, Joan T. Merrill

**Affiliations:** ^1^Department of Clinical Sciences and Community Health, University of Milan, Milan, Italy; ^2^Division of Rheumatology, Lupus Clinic, Istituto Ortopedico Gaetano Pini, Milan, Italy; ^3^Department of Neurology-Stroke Unit and Laboratory of Neuroscience, IRCCS Istituto Auxologico Italiano, Milan, Italy; ^4^Experimental Laboratory of Immunological and Rheumatologic Researches, IRCCS Istituto Auxologico Italiano, Milan, Italy; ^5^Allergy, Clinical Immunology and Rheumatology Unit, IRCCS Istituto Auxologico Italiano, Milan, Italy; ^6^Department of Pathophysiology and Transplantation, “Dino Ferrari” Center, Università degli Studi di Milano, Milan, Italy; ^7^Clinical Pharmacology Research Program, Oklahoma Medical Research Foundation, University of Oklahoma, Oklahoma City, OK, USA

**Keywords:** systemic lupus erythematosus, antiphospholipid syndrome, mild cognitive impairment, neuropsychological assessment, central nervous system involvement, anti-NMDA/glutamate receptor antibodies

## Abstract

Systemic lupus erythematosus (SLE) and antiphospholipid syndrome have an increased risk to develop cognitive impairment. A possible role for antiphospholipid antibodies (aPL) and antiglutamate receptor (anti-NMDA) antibodies in the pathogenesis of neurological manifestations of these two conditions, have been suggested. In particular, the role of anti-NMDA antibodies in the pathogenesis of neuropsychiatric SLE is supported by several experimental studies in animal models and by the finding of a correlation between anti-NMDA positivity in cerebrospinal fluid and neurological manifestations of SLE. However, data from the literature are controversial, as several studies have reported a correlation of these antibodies with mild cognitive impairment in SLE, but more recent studies have not confirmed this finding. The synergism between anti-NMDA and other concomitant autoantibodies, such as aPL, can be hypothesized to play a role in inducing the tissue damage and eventually the functional abnormalities. In line with this hypothesis, we have found a high incidence of at least one impaired cognitive domain in a small cohort of patients with primary APS (PAPS) and SLE. Interestingly, aPL were associated with low scoring for language ability and attention while anti-NMDA titers and mini-mental state examination scoring were inversely correlated. However, when patients were stratified according to the presence/absence of aPL, the correlation was confirmed in aPL positive patients only. Should those findings be confirmed, the etiology of the prevalent defects found in PAPS patients as well as the synergism between aPL and anti-NMDA antibodies would need to be explored.

## Introduction

Systemic lupus erythematosus (SLE) is a chronic disease characterized by antibodies directed to different components of the cell nucleus in association with a variety of clinical manifestations, including skin rash, arthritis, serositis, nephritis, hematological cytopenias, and neurological manifestations. The prevalence ranges from 15 to 50 cases per 100,000 persons and the incidence is around 2–8 new cases per 100,000 persons per year. Polyclonal B-cell stimulation and autoantibody production, leading to immune complex deposition and complement activation represent the most important pathogenic mechanisms of the disease (1). Treatment of SLE largely depends on the type of clinical manifestations: antimalarial agents in association with low-dose steroids represent the first choice in mild disease, while immunosuppressant agents, such as azathioprine, methotrexate, cyclophosphamide, and mycophenolate mofetil are used in more severe manifestations. Recently, belimumab, a new biological agent able to modulate B lymphocyte function, has been demonstrated to reduce lupus disease activity (1).

Antiphospholipid syndrome (APS) is a systemic autoimmune disease close to SLE with significant overlap of serological and clinical characteristics (2). It is mainly characterized by pregnancy complications and thrombotic events, involving both the venous and the arterial district (2). The formal classification of APS requires the persistent presence of medium to high titers of antiphospholipid antibodies (anti-PL), namely anti-β2 glycoprotein I (anti-β2GPI), anticardiolipin (anti-CL), and lupus anticoagulant (LA) (2). The disease can occur as an isolate clinical entity [primary APS (PAPS)] or associated with other autoimmune diseases, mainly with SLE (secondary APS). The prevalence is estimated around 40–50 cases per 100,000 persons and the incidence is around five new cases per 100,000 persons per year. Elevated levels of anti-PL and positivity to more than one test have been linked to a higher risk for developing the disease (3). Treatment of APS is based on prevention of recurrence and is mainly represented by long term anticoagulation. Hydroxychloroquine, statins, rituximab, and eculizumab can be considered in refractory cases (3).

## Cognitive Impairment in APS and SLE

Among the several neurological symptoms related to SLE and classified by the American College of Rheumatology (3), cognitive dysfunctions have been reported to affect 6–66% of the patients according to different studies, but the prevalence may be up to 95% when cognitive defects are assessed by computerized neuropsychological testing (4). This literature is confusing, and the real incidence of dementia or mild cognitive defects in SLE patients has not yet been well defined.

Neurological involvement is very frequent in APS, cerebral stroke being one of the most common vascular manifestations. In fact, ischemic events are the most frequently observed central nervous system (CNS) complications of PAPS and represent one of the formal classification criteria. Multi-infarct dementia is also described in these patients, mainly associated with recurrent ischemic events, with an incidence estimated as 10–56% increasing with age (3, 5, 6). Additional manifestations, such as migraine, seizures, chorea, transverse myelopathy, and multiple sclerosis-like syndrome have been reported (3). Cognitive impairment has frequently been addressed as a neurological APS “non-classification criterion.” However, this finding has usually been described in APS associated with an underlying systemic autoimmune disease in the majority of the reports, mainly in SLE-associated APS, while only two studies are available in the literature in PAPS (5, 6). A case–control study of 60 patients with primary or secondary APS found that 42% presented mild cognitive defects, primarily involving verbal fluency and attention. Looking at PAPS patients only, the prevalence was about the same (38%); however, besides anti-PL, no further correlation with serological profiles was evaluated (5). Similar results have recently been reported in a study comparing the cognitive function of anti-PL positive non-SLE subjects and anti-PL negative SLE patients (6). In this study, 20 SLE anti-PL negative patients and 20 anti-PL positive subjects, with or without clinical manifestations of APS, were investigated through a standardized cognitive test battery; 60% of SLE and 40% of anti-PL positive subjects displayed a cognitive impairment assessed by a global cognitive impairment index (CCI). This finding was not associated to disease activity or duration, while a possible correlation with the whole serological profile was not assessed (6).

Cognitive defects in PAPS cannot be simply explained by ischemic events; therefore, a direct effect of anti-PL on neuronal cells was suggested. Accordingly, anti-PL were found to bind CNS cells *in vitro* (7) and the intrathecal passive transfer of class G immunoglobulins (IgG) from APS patients were shown to induce cognitive defects in mice *in vivo* (7). These results have been recently confirmed by the same group, which demonstrated that the intracerebroventricular (ICV) passive transfer of human anti-PL IgG purified from the serum of APS patients with CNS involvement induced specific hyperactivity in injected mice (7). In addition to anti-PL, PAPS patients can display several organ non-specific autoantibodies, such as ANA, anti-dsDNA, antinucleosome, and anti-extractable nuclear antigen (anti-ENA), even in the absence of evident clinical manifestations of SLE (3).

Lupus-specific mechanisms underlying neuropsychiatric disease are better known and have been related to vasculitides of intracranial vessels, local or systemic production of inflammatory mediators, and generation of specific autoantibodies (8). Different types of autoantibodies have been investigated, such as aPL themselves and antiribosomal P protein antibodies (4, 8, 9). An increasing interest has been raised during the last decade for antibodies reacting to the human glutamate receptor (anti-NMDA) as potentially involved in the pathogenesis of neurological manifestations of SLE.

## Anti-NMDA Antibodies

A murine monoclonal anti-DNA antibody has been recently shown to cross-react with the extracellular domain of mouse and human glutamate receptor (NMDA) and to induce cognitive dysfunction in mice through neuronal cell apoptosis (10). Affinity-purified anti-NMDA antibodies were demonstrated to induce apoptotic cell death *in vitro*, when added to neuronal cultures. Moreover, mice immunized with a specific antigen to produce anti-NMDA antibodies developed cognitive dysfunction and spatial memory impairment only when a blood–brain barrier (BBB) disruption occurred (10). The autoantibodies selectively bound to hippocampal neurons and caused neuronal death. Interestingly, the administration of memantine, an NMDA receptor antagonist, prior to BBB disruption, prevented neuronal damage (10). Moreover, the direct injection of antibodies eluted from the brain of a SLE patient with severe CNS involvement in the C57BL/6 mice hippocampus was able to induce neuronal loss (10). More recently, the same group has demonstrated that anti-NMDA antibodies development was associated with selective impairment of spatial memory in female BALB/c mice and that relevant structural abnormalities could be observed in the surviving hippocampal pyramidal neurons (11). Further investigations have shown the presence of similar anti-NMDA antibodies in both serum and cerebrospinal fluid (CSF) of SLE patients even if a clear clinical association between these autoantibodies and cognitive defects has not yet been established (12).

Anti-NMDA antibodies have been reported in SLE patients with a prevalence of 19–33% in different studies (4, 9). Given their high pathogenicity of these in experimental animal models, a possible relationship with neuropsychiatric manifestations of SLE was investigated. Some authors have reported an association between anti-NMDA and several neurological manifestations, such as cognitive impairment, decline in memory functions, impaired attention or executive functions, and depression. However, some other studies in very large cohorts of lupus patients have not confirmed these data (4, 9). Remarkably, an association between the presence of anti-NMDA in the CSF and NP-SLE (neuropsychiatric SLE) manifestations was demonstrated in different studies. Moreover, CSF anti-NMDA titers correlated with clinical manifestations and with evidence of BBB dysfunction (11). Data examining the PAPS population for anti-NMDA are scant. No correlation between the presence of anti-NMDA and anti-CL in a SLE population was previously found, but these autoantibodies have not been evaluated in selected cohorts of primary APS patients (4).

**Figure 1:**
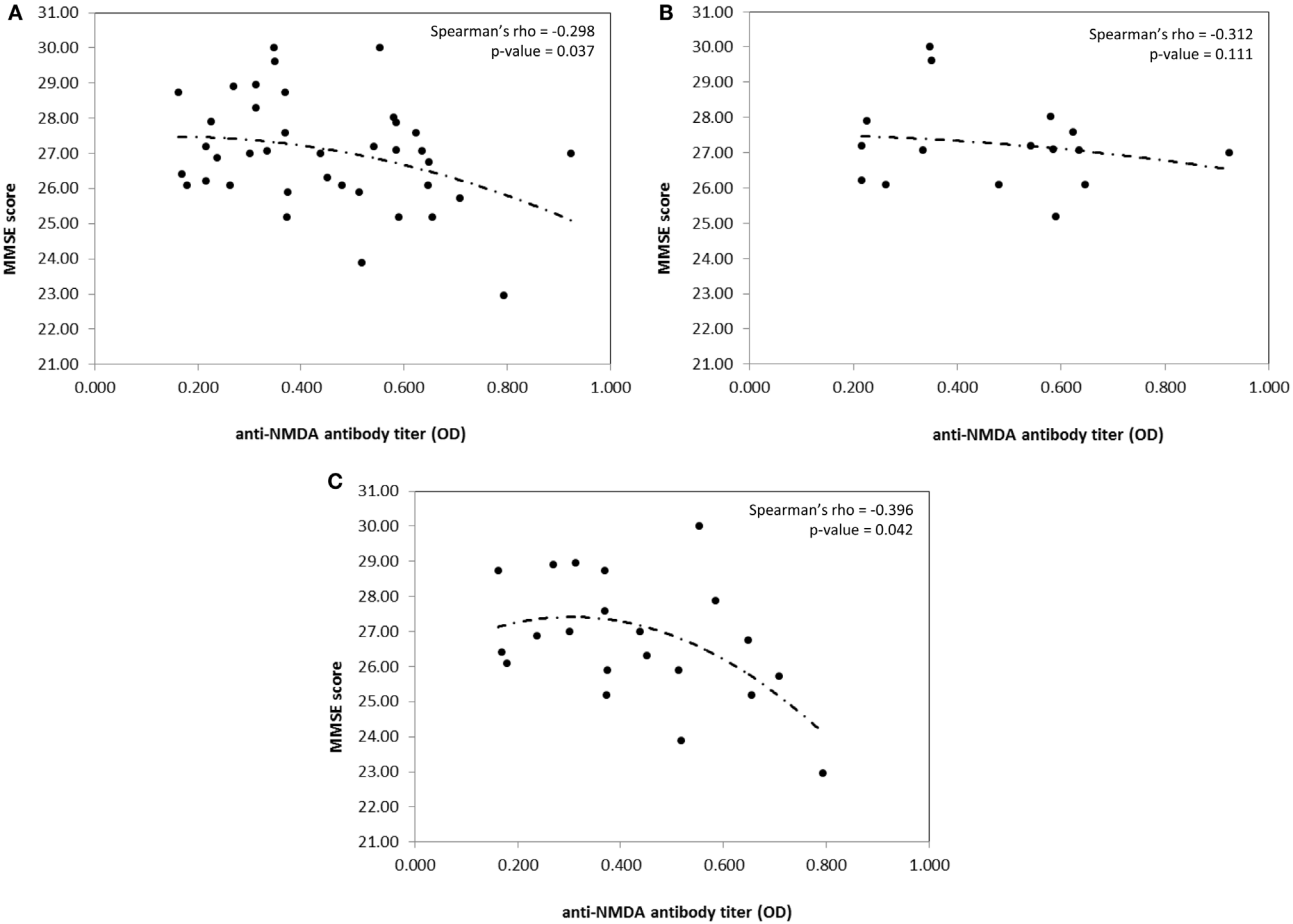
**Correlation between MMSE score and anti-NMDA antibody titer in the whole population (A), aPL negative patients (B), and aPL positive patients (C), only**. Spearman’s rho coefficient and relative *p*-value are reported on the top right corner of the graph. Curves are fitted according to a second polynomial equation model.

With this as a background, we have recently examined the incidence of cognitive dysfunctions in a cohort of consecutive unselected PAPS in order to compare the results with those found in a parallel series of SLE patients and to better characterize the pattern of cognitive involvement in these two diseases and the possible associations between cognitive defects and the autoantibody profile. The protocol was approved by the Institutional Review Board (IRB) and informed consent was obtained from all the participants. Forty-two patients, 15 with a diagnosis of PAPS and 27 with a diagnosis of SLE, regularly followed in our Unit, underwent a 180 min neuropsychological test battery. Anti-NMDA levels were detected by a home-made enzyme-linked immunosorbent assay (ELISA) using a branched peptide as antigen (Asp Trp Glu Tyr Ser Val Trp Leu Ser Asn8 Lys4 Lys2 Lys-β Ala) (9). Among SLE patients, all were ANA positive, three were positive for antiribosomal P antibodies, 13 for anti-dsDNA antibodies at medium/high titer, eight for LA, three for anti-CL IgG, five for anti-β_2_GPI IgG, two for anti-CL IgM, and three for anti-β_2_GPI IgM at medium/high titer. Among PAPS patients, ANA were positive in 8 and 2 were positive for medium/high titer anti-dsDNA antibodies. All patients had high titer anti-CL IgG and 14 patients high titer anti-β_2_GPI IgG, while LA was positive in 13 patients. Anti-NMDA antibodies were positive in 12 SLE and 5 PAPS, with no differences in prevalence and titer range. Considering the whole population, the incidence of cognitive dysfunctions was high since 76% of the population (13 PAPS and 19 SLE) exhibited at least one defective domain. In five PAPS and three SLE patients, four or more tests were pathologic and mathematical abilities, attentional skills, and visual-motor planning were the most frequently affected cognitive domains. However, when comparing score severity, PAPS patients significantly deviate from SLE patients in the Phonemic Verbal fluency (*U* = 121.5; *p* = 0.018) and Street’s Completion Test (*U* = 128.0; *p* = 0.043). The presence of anti-PL was found to correlate with lower scoring for language ability and attention. A correlation between high anti-NMDA IgG titers and low scoring for the mini-mental state examination (MMSE) was found in the whole population (Rho −0.298; *p* = 0.037). However, when patients were stratified according to the presence or the absence of anti-PL, the correlation was maintained in anti-PL positive patients only (Rho – 0.396; *p* = 0.042) (Figure 1). Higher anti-NMDA titers were associated with low scores for the MMSE test, independently from the disease diagnosis. In Table 1 are summarized the results of the most important studies regarding anti-NMDA in SLE.

**Table 1:** **Summary of the studies on anti-NMDA in SLE**.

			Reference
*In vitro* experiments	• Anti-NMDA ab from SLE pts cross-react with anti-dsDNA • Affinity-purified anti-NMDA ab added to neuronal cultures induce apoptotic cell death • Anti-NMDA/dsDNA cross-reactive ab induce “excitotoxicity” and neuronal apoptosis		DeGiorgio et al. (13), Kowal et al. (14), Kowal et al. (10), Chang et al. (11)

Humans	• Anti-NMDA ab have been detected in 14–35% of SLE patients^a–f^ • No correlation between the presence of anti-NMDA and anti-CL in a SLE population^f^ • Some authors have reported an association between anti-NMDA and Cognitive impairment^a^ Decline in memory functions^a^ Impaired attention or executive functions^a^ Depression^a,b^ NPSLE^g^ •Recent studies in very large cohorts of lupus patients have reported NO association between anti-NMDA with Cognitive impairment^b–d,h^ Epilepsy^i^ Mood disorders^c,d^ •Several studies have demonstrated an association between the presence of anti-NMDA in the CSF and NPSLE^h–l^ •CSF anti-NMDA titers correlated with clinical manifestations and with evidence of BBB dysfunction^h–l^		a. Omdal et al. (15) b. Lapteva et al. (16) c. Harrison et al. (17) d. Hanly et al. (9) e. Kozora et al. (18) f. Husebye et al. (19) g. Gono et al. (20) h. Hanly et al. (21) i. Arinuma et al. (22) j. Yoshio et al. (23) k. Hirohata et al. (12) l. Chang et al. (11)

Our results	• 27 SLE and 15 PAPS underwent an extensive neuropsychological text battery • 44% SLE and 33% PAPS anti-NMDA ab positive (no differences in prevalence and titer) • 70% SLE and 86% PAPS exhibited at least 1 defective domain • 11% SLE and 33% PAPS exhibited 4 or more pathologic tests • Mathematical abilities, attentional skills, and visual-motor planning were the most frequently affected cognitive domains • PAPS patients significantly deviate from SLE patients in the Phonemic Verbal fluency and Street’s Completion Test • Anti-PL correlate with lower scoring for language ability and attention • High anti-NMDA IgG titers correlate with low scoring for MMSE in the whole population and in anti-PL-positive patients but NOT in aPL-negative patients independently from diagnosis		

Animal models	• BALB/c mice immunization to produce anti-NMDA antibodies or • Intravenous infusion of serum from SLE pts, containing anti-NMDA ab in BALB/c mice • BBB abrogation induced by LPS	Cognitive dysfunction and spatial memory impairment development Neuronal binding and apoptotic death in hippocampus Prevention of antibody-mediated injury by the NMDA receptor antagonist memantine	DeGiorgio et al. (13), Kowal et al. (14)

	• BALB/c mice immunization to produce anti-NMDA antibodies^n^ • BBB abrogation induced by epinephrine^o^	Neuronal binding and apoptotic death in amygdala^n^ Aberrant pavlonian fear conditioning^o^	n. Kowal et al. (14) o. Huerta et al. (24)

	• Direct injection of ab eluted from the brain of a severe NPSLE patient in the C57BL/6 mice hippocampus	ab-mediated neuronal damage	Kowal et al. (10)

*Ab, antibodies; pts, patients; BBB, blood–brain barrier; LPS, lipopolysaccharide; NPSLE, neuropsychiatric SLE; CSF, cerebrospinal fluid; MMSE, mini-mental state examination*.

## Comments

Systemic lupus erythematosus patients are known to be more likely to develop cognitive impairment compared to the general population, and an association with anti-PL or anti-NMDA antibodies has been suggested (4, 5). Data from the literature are controversial, as several studies have reported a correlation of these antibodies with mild cognitive impairment in SLE, but more recent studies have not confirmed this finding. The role of anti-NMDA antibodies in the pathogenesis of NP-SLE is supported by experimental studies and by the finding of a correlation between anti-NMDA positivity in CSF and neurological manifestations of SLE. Not all the anti-NMDA positive patients display a BBB interruption that can allow circulating antibodies to reach the CNS and induce damage. Moreover, patients with previous BBB damage could recover, limiting access to the brain for the autoantibodies, which might continue to be measured in the peripheral blood. In this regard, the synergism between anti-NMDA and other concomitant autoantibodies could play a role in inducing the tissue damage and eventually the functional abnormalities. In line with this hypothesis, we can speculate that one of the potential mechanisms leading to BBB disruption could be represented by the endothelial perturbation induced by β2GPI-dependent aPL, which could possibly facilitate the access of anti-NMDA antibodies in the cerebral circulation. Nevertheless, infections, CNS inflammation, or other autoantibodies interaction can alternatively be implicated in BBB damage.

Our preliminary results are in accordance with this assumption. The occurrence of anti-NMDA antibodies in PAPS patients is not surprising. Anti-NMDA activity was originally described in anti-DNA antibodies derived from SLE mice (10) and antibodies to nuclear antigens can be found in PAPS (3).

In conclusion, both anti-PL and anti-NMDA antibodies behave as promising potential biomarkers of CNS involvement even if further longitudinal studies are essential to define the risk for a potential evolution and to also clarify the opportunity of starting a possible preventive therapy. Due to the great impact of cognitive impairment on quality of life and given the cognitive decline which has been described even in patients with a recent diagnosis, we propose that SLE and PAPS patients should be monitored for the development of cognitive impairment.

## Author Contributions

MG, BP, PR, and PM gave a substantial contribution to the conception and design of the work and interpretation of data. FP, AL, GC, VS, and JM gave a substantial contribution to acquisition, analysis, and interpretation of data. MG, BP, and PM participate in drafting the work. FP, GC, AL, PR, VS, and JM revised it critically for important intellectual content. MG, BP, FP, AL, GC, VS, PR, PM, and JM gave their final approval of the version to be published; and agreed to be accountable for all aspects of the work in ensuring that questions related to the accuracy or integrity of any part of the work are appropriately investigated and resolved.

## Conflict of Interest Statement

The authors declare that the research was conducted in the absence of any commercial or financial relationships that could be construed as a potential conflict of interest.

## Abbreviations

ANA, antinuclear antibodies; anti-CL, anticardiolipin; anti-dsDNA, antidouble strand DNA; anti-PL, antiphospholipid antibodies; anti-β2GPI, anti-β2 glycoprotein I; APS, antiphospholipid syndrome; BBB, blood–brain barrier; CCI, cognitive impairment index; CNS, central nervous system; CSF, cerebrospinal fluid; ELISA, enzyme-linked immunosorbent assay; ENA, extractable nuclear antigen; ICV, intracerebroventricular; Ig, immunoglobulins; LA, lupus anticoagulant; MMSE, mini-mental state examination; NMDA, human glutamate receptor; NPSLE, neuropsychiatric SLE; PAPS, primary APS; SLE, systemic lupus erythematosus.
